# A Deconvolution Protocol for ChIP-Seq Reveals Analogous Enhancer Structures on the Mouse and Human Ribosomal RNA Genes

**DOI:** 10.1534/g3.117.300225

**Published:** 2017-11-20

**Authors:** Jean-Clement Mars, Marianne Sabourin-Felix, Michel G. Tremblay, Tom Moss

**Affiliations:** * Laboratory of Growth and Development, St-Patrick Research Group in Basic Oncology, Cancer Division of the Quebec University Hospital Research Centre, G1R 3S3, Canada; † Department of Molecular Biology, Medical Biochemistry and Pathology, Faculty of Medicine, Laval University, Québec, Canada

**Keywords:** ChIP-Seq deconvolution, RNA polymerase I (RPI, PolI, Polr1), ribosomal RNA (rRNA) genes, upstream binding factor (UBF/UBTF), selectivity factor SL1

## Abstract

The combination of Chromatin Immunoprecipitation and Massively Parallel Sequencing, or ChIP-Seq, has greatly advanced our genome-wide understanding of chromatin and enhancer structures. However, its resolution at any given genetic locus is limited by several factors. In applying ChIP-Seq to the study of the ribosomal RNA genes, we found that a major limitation to resolution was imposed by the underlying variability in sequence coverage that very often dominates the protein–DNA interaction profiles. Here, we describe a simple numerical deconvolution approach that, in large part, corrects for this variability, and significantly improves both the resolution and quantitation of protein–DNA interaction maps deduced from ChIP-Seq data. This approach has allowed us to determine the *in vivo* organization of the RNA polymerase I preinitiation complexes that form at the promoters and enhancers of the mouse (*Mus musculus*) and human (*Homo sapiens*) ribosomal RNA genes, and to reveal a phased binding of the HMG-box factor UBF across the rDNA. The data identify and map a “Spacer Promoter” and associated stalled polymerase in the intergenic spacer of the human ribosomal RNA genes, and reveal a very similar enhancer structure to that found in rodents and lower vertebrates.

Data from Chromatin Immunoprecipitation (ChIP) combined with Massively Parallel DNA Sequencing (ChIP-Seq) can potentially provide high-resolution maps of transcription and chromatin factor interactions throughout the genome. The absolute resolution of these maps is determined by the size-range of chromatin fragments that are selected during the ChIP step. However, in practice, several other factors limit the resolution achieved by the technique. These include the relative accessibility of the targeted protein–DNA complex (Teytelman *et al.* 2013), the efficiency of crosslinking, the combined effects of these limitations on complex recovery (Poorey *et al.* 2013), and the selectivity of the ChIP step. But a major limitation to mapping resolution is also imposed by the strong biases in DNA sequence coverage inherent in the Seq protocols. Sequence coverage biases have previously been noted for mitochondrial DNAs, and shown to correlate with DNA composition and certain sequence motifs (Ekblom *et al.* 2014). Several data normalization approaches have been developed to correct for biases in sequence coverage maps (Park 2009; Kidder *et al.* 2011; Chen *et al.* 2012; Taslim *et al.* 2009), but are predominantly aimed at improving the reliability of the peak calling routines used to identify potential factor binding sites genome-wide, and have had only limited success (Teytelman *et al.* 2013). However, when investigating details of factor binding at given sites within the genome, these approaches fail to correct for local biases in sequence coverage, and hence do little to improve mapping resolution of complexes at specific DNA sites.

Here, we show that a simple numerical deconvolution approach successfully removes the sequencing biases introduced into ChIP-Seq data by Seq techniques, and greatly improves the resolution of protein–DNA interaction maps. We have applied this approach to better understand the structure of the duplicated RNA polymerase I (RPI/PolI) promoters, preinitiation complexes and enhancers that form on the ribosomal RNA genes (rDNA) of mouse and human. Duplications of RPI promoters are found within the rDNA Intergenic Spacers (IGS) of insects, amphibia, and rodents, and are often referred to as “Spacer Promoters”. They were first identified in the rDNA IGS of *Xenopus laevis* (Moss and Birnstiel 1979) and of *Drosophila melanogaster* (Coen and Dover 1983; Miller *et al.* 1983), but later were also found in other *Xenopus* and *Drosophila* species, and in mouse, Chinese hamster, rat, and even plants (Bach *et al.* 1981; Murtif and Rae 1985; Kuhn and Grummt 1987; Tower *et al.* 1989; Cassidy *et al.* 1987; Doelling *et al.* 1993). These Spacer Promoters function as part of upstream transcriptional enhancer elements (Moss 1983; De Winter and Moss 1986, 1987; Paalman *et al.* 1995; Caudy and Pikaard 2002), and are often repeated several times within a given IGS (reviewed in Moss *et al.* 1985, 2007; Moss and Stefanovsky 1995). More recently, the mouse Spacer Promoter has been suggested to be the source of a long noncoding RNA (lncRNA) that is responsible for *in trans* silencing and heterochromatinization of the rDNA and centric and pericentric chromosomal repeats (Guetg *et al.* 2010; Savic *et al.* 2014). But, despite their demonstrated importance in transcription and silencing, the mouse and rat Spacer Promoters remain only partially mapped, while the existence of Spacer Promoters in other mammals, and even in humans, is still largely a matter of speculation. Our deconvolution protocol revealed significant *in vivo* detail of the RPI or PolI preinitiation complexes that form at the functional 47S rRNA gene promoters and the Spacer Promoters in mouse, and showed that they are indistinguishable, despite the very poor homology between the underlying DNA sequences. The deconvolution protocol further identified and mapped a Spacer Promoter in the human rDNA IGS, and showed that it exists in the context of an enhancer complex closely resembling that occurring in mouse.

## Materials and Methods

### ChIP

Cells were fixed with 1% formaldehyde for 8 min at room temperature. Nuclei were isolated using Lysis Buffer (10 mM Tris, pH 7.5, 10 mM NaCl, 3 mM MgCl_2_, 0.5% NP-40), transferred to Sonication Buffer (50 mM Tris-HCl, pH 7.5, 150 mM NaCl, 2 mM EGTA, 4 mM EDTA, 0.1% SDS, 1% Triton X-100, 1% NP-40) and sonicated (Bioruptor; Diagenode) for 30 cycles of 30 sec on/30 sec off at high intensity. Each immunoprecipitation (IP) was carried out on the equivalent of 50 × 10^6^ cells in IP Buffer (150 mM NaCl, 50 mM Tris-HCl pH 7.5, 5 mM EDTA, 0.5% NP-40, 1% Triton X-100) overnight at 4°. The antibody slurry was prepared with 50 µl A-, 50 µl G-Dynabeads, and 60 µg ml^−1^ antibody per IP. Immunoprecipitated chromatin was treated with RNaseA and the DNA isolated using 2% Na SDS and 2 mg ml^−1^ Proteinase-K. Two or more biological replicates were analyzed for each antibody.

### Analysis of ChIP samples by massively parallel sequencing

ChIP DNA samples were quality controlled by qPCR as previously described (Herdman *et al.* 2017), before being sent for library preparation and 50 base single-end sequencing on an Illumina HiSequation 2000 by Genome Quebec (McGill University and Genome Quebec Innovation Centre).

### ChIP-seq data alignment

The raw fastq.gz files from ChIP and input DNA were checked for quality using FastQC version 0.11.4 (Babraham Bioinformatics, S. Andrews). The data were then trimmed using Trimmomatic version 0.33 (Bolger *et al.* 2014) with the following parameters: LEADING:32, TRAILING:32, MINLEN:36, ILLUMINACLIP:TruSeq3-SE.fa:2:30:10. The resulting trimmed files were aligned to modified versions of the mouse and human genomes using Bowtie2 (Langmead and Salzberg 2012) with option −*k 3*. Alignment of the mouse data were to the mouse genome version GRCm38, to which a single copy of the rDNA repeat sequence (GenBank BK000964v3) was added as an extra chromosome. For convenience, the origin of the rDNA repeat was displaced to the *Eco*RI site at 30,493 such that the pre-rRNA initiation site now fell at nucleotide 14,815.

Alignment to the human rDNA proved a little more difficult using the same strategy due to the multiple rDNA sequences already present in version GRCh38. We therefore first searched the human *in silico* genome for regions most likely to interfere with alignment of rDNA sequences. The “canonical” rDNA repeat sequence (GenBank accession number U13369.1) was fragmented to generate 50 bp nonoverlapping pseudoreads, and these aligned on GRCh38 using Bowtie2 with the −*k 10* option. This identified three major regions that would interfere with ChIP-Seq data alignment. The reference genome was, therefore, modified to remove these occurrences; the chromosomes chr22_KI270733v1_random and chrUn_GL000220v1 were removed, and the rDNA sequence present on chromosome 21 was replaced with N (8,202,082–8,552,360). A single copy of the human rDNA repeat (GenBank accession number U13369.1) was then added as an extra chromosome. For convenience, the origin of the rDNA sequence was moved to the *Eco*RI site at 30,487, such that the pre-rRNA initiation site now fell at nucleotide 12,514.

### Deconvolution protocol

The rDNA chromosome was first extracted from the aligned file with the *view* command of SAMtools (Li *et al.* 2009). The rDNA data were then converted from BAM to BED6 format using the *bamtobed* command of the BEDTools suite version 2.25.0 (Quinlan and Hall 2010). Each read was extended 3′ to the mean fragment length computed using the *makeTagDirectory* command of HOMER v4.3 (Heinz *et al.* 2010). Estimated fragment lengths fell between 75 and 125, and so were standardized to the mean size of 100 bp. The coverage was then extracted with the *genomecov* command of BEDTools, smoothed using a 25-bp sliding window, and adjusted to reads per million (RPM). Data deconvolution was achieved by dividing the calculated sample DNA coverage by the appropriate input DNA coverage in order to remove the sequence coverage biases introduced by the sequencing protocol, as described in the main text. At positions where coverage in either data set was of low statistical significance, the deconvoluted data were set to 0 and ignored in subsequent interpretations. The resulting deconvoluted ChIP-Seq data were converted to BedGraph format and visualized using IGV (Integrative Genomics Viewer 2.3; Broad Institute). The manual for the deconvolution protocol and a corresponding Python script can be found at https://github.com/mariFelix/deconvoNorm. Gaussian curve fitting to rDNA promoter subregions was perform using MagicPlot Pro (Magicplot Systems) on data extracted from the BedGraph files.

### Alignment of ChIP-nexus data

The 5′ ends of reads from the ChIP-nexus datasets were mapped by first aligning sequences using Bowtie2 as above, but using the unique mapping −*k 1* option. A Bedgraph of coverage for the 5′ position of each aligned read was then extracted using the *genomecov* command of BEDTools with the parameters –5, and –strand + (for forward reads) or –strand − (for reverse reads), and visualized using IGV.

### Data availability

Mouse strains are available from Jackson Laboratories (JAX Stock No. 029470, Ubtf < tm1.1Tmss>/J), and a very limited supply of derived cell lines may also be available upon request. Human cell lines are available from ATCC. The mouse mapping data can be found on ArrayExpress under the accession number E-MTAB-5839. The human data for UBF and RPI in K562 cells can be found on ArrayExpress under the accession number E-MTAB-6032. The HEK293T (UBF, RPI, and input) and K562 (UBF and input) data from Zentner *et al.* (2011) can be found on the SRA database under the accession number SRP004897. The K562 data (UBF, TBP, and input) from ENCODE can be found on the GEO DataSets database under the accession number GSE31477. The K562 data (CTCF and input) from ENCODE can be found on the GEO DataSets database under the accession numbers GSE29611 and GSE70764. The ChIP-exonuclease data for TBP can be found on the GEO DataSets database under the accession number GSE55306. A manual for the deconvolution protocol, a corresponding Python script, and sample datasets can be found at https://github.com/mariFelix/deconvoNorm.

## Results

**Figure 1:**
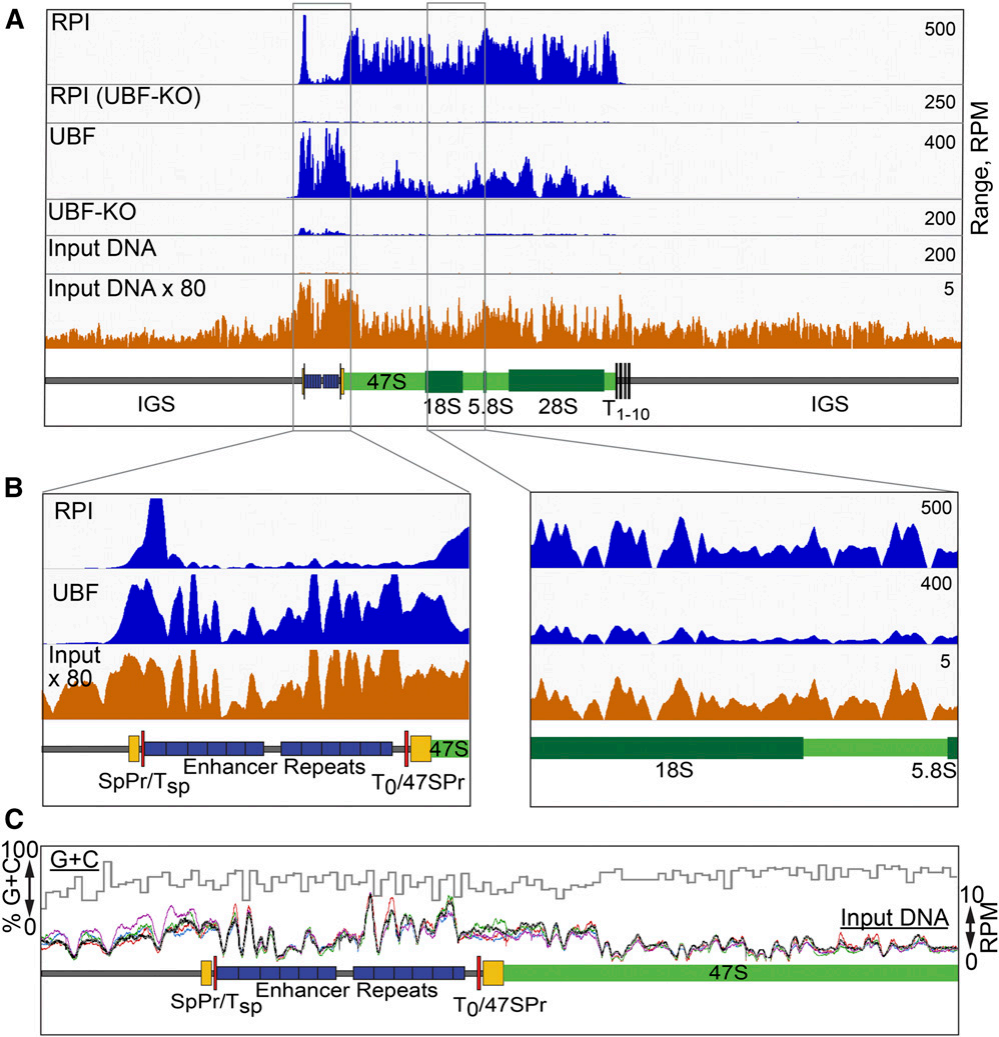
Sequence coverage dominates the raw ChIP-Seq profiles for UBF and RPI. (A and B) Comparison of the ChIP-Seq profiles for RPI and UBF with the sequencing coverage for unselected “input” DNA. UBF and RPI ChIP-Seq data after UBF knock out (UBF-KO) is shown to demonstrate the specificity of the respective antibodies used. Diagrammatic maps of the rDNA are given below the mapping profiles, showing the 47S transcribed region and the 18S, 5.8S, and 28S genes in green, the enhancer repeats in blue, the extents of the 47S and known Spacer Promoters (47SPr, SpPr) in yellow, and the TTF1 binding sites T_sp_, T_0_ and T_1–10_ in red. Coverage across the complete rDNA repeat is shown in (A) and enlargements across the enhancer and the central 47S transcribed regions in (B). The vertical scales in (A and B) are given in RPM. (C) A superimposition of sequence coverage in RPM for five biological replicas of unselected “input” DNA is shown below the percent G+C sequence composition across the upstream region of the rDNA.

In order to better understand the *in vivo* functions of the RPI transcription factors, as part of an extensive study (Herdman *et al.* 2017), we performed ChIP analysis of wild type and conditional mouse embryonic fibroblasts using antibodies specific for the various factors, and subjected the resulting DNA fragments to Seq. The raw data were quality checked and trimmed and then aligned to the digital mouse genome that included a single rDNA repeat using Bowtie2, see *Materials and Methods*. Examples of the resulting factor binding profiles are shown in Figure 1A.

When mapping RPI/PolI engagement across the mouse rDNA gene body by ChIP-Seq, we expected to observe the dense, relatively even distribution of RPI seen in electron-microscope images of single mouse rRNA genes (Scheer and Benavente 1990). In contrast, the ChIP-Seq coverage maps suggested an extremely uneven distribution of RPI (Figure 1A), as had been previously noted in human (Zentner *et al.* 2011). This was even more surprising considering that the ChIP technique should reveal the summed RPI distribution across the several hundred active rRNA gene copies in each cell as averaged over a population of many millions of cells. Similarly, sequence coverage maps for the multi-HMGB-box factor UBF (UBTF) also suggested very variable occupancy across the gene (Figure 1A).

### ChIP-Seq profiles result from a convolution of the protein crosslinking and sequencing coverage profiles

ChIP of both UBF and RPI was extremely specific, since conditional inactivation of the floxed UBF gene (UBF-KO) in MEFs strongly suppressed sequence enrichment when using antibodies against either factor, RPI engagement being dependent on UBF (Hamdane *et al.* 2014; Herdman *et al.* 2017) (Figure 1A). Strikingly, both the RPI and UBF sequence coverage profiles displayed a strong similarity to the coverage distribution obtained for unselected (input) genomic DNA from the same chromatin preparations. This similarity was clearly apparent when sequence coverage was compared at higher resolution (Figure 1B). Both in the case of RPI and UBF, the ChIP-Seq profiles closely followed the input DNA sequence profiles over the same regions. Hence, the RPI and UBF interactions profiles were clearly superimposed on a pattern resulting from the unevenness of sequence coverage, and, indeed, this pattern dominated these interaction profiles. However, we noted that the pattern of input DNA sequence coverage was highly reproducible between biological preparations (Figure 1C). Thus, it was clearly a property intrinsic to the Seq protocol, and did not result from variations in sample preparation. But, unlike the bias in sequence coverage observed for mitochondrial DNA (Ekblom *et al.* 2014), we saw little, if any, correlation with the local rDNA GC content (Figure 1C). The coefficient of determination *R*^2^ between the mean input read profile of the five datasets shown and the GC content, both determined over 25-bp windows, was 0.07 for the full rDNA repeat and 0.002 for the 47S transcribed region.

### Deconvolution of ChIP-Seq data provides greatly improved resolution in protein–DNA interaction maps

**Figure 2:**
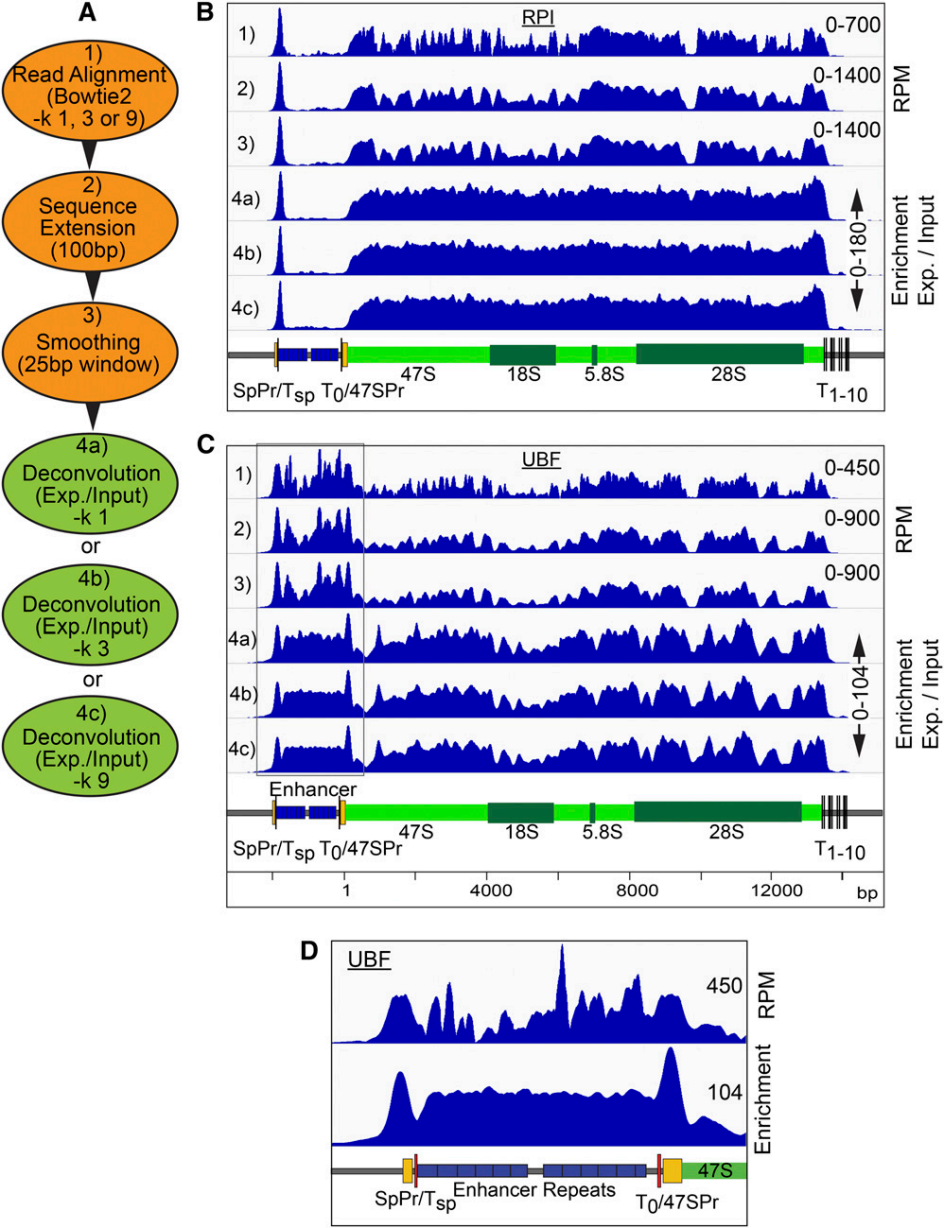
Improved mapping with ChIP-Seq deconvolution. (A) Summary of ChIP-Seq data handling, steps 1–3, and deconvolution, step 4. (B and C) Examples of the sequence coverage maps across the mouse rDNA at each step, 1–4, of data treatment respectively for RPI and UBF ChIP-Seq. (D) Comparison of UBF mapping over the upstream gene region before [as in (C) lane 1], and after [as in (C) lane 4b] deconvolution. In (B–D), the vertical scales are given either in RPM or as enrichment relative to input DNA, and diagrammatic maps of the rDNA are given below the mapping profiles.

The reproducibility of input sequence coverage profiles suggested that it should be possible to remove these sequencing biases by numerical deconvolution. However, despite average input DNA sequencing depths of well over 100, initial attempts at deconvolution by directly normalizing the raw sample to input (*sample coverage/input coverage*) for each base position gave an unacceptable level of noise in the mapping profile. To counter this without significantly affecting mapping resolution, we incorporated two steps prior to deconvolution (Figure 2A). Sequences were first extended to the predicted DNA fragment length, then sequence coverage was smoothed using a sliding window, see examples for RPI and UBF (tracks 1–3, Figure 2, B and C). DNA fragment lengths were estimated using HOMER (Heinz *et al.* 2010) and found to consistently fall between 75 and 125 bp. Thus, for convenience DNA fragment sizes of all sample and input data sets were standardized to the mean size of 100 bp. We also investigated smoothing using three sizes of sliding window “w” (11, 25, or 51 bp), such that:

$$\text{Smoothed}\;\text{base}\;\text{coverage}\;J=\frac{1}{w}•\sum_{n+\left(w-1\right)/2}^{n-\left(w-1\right)/2}j_{n},$$

where *j* = aligned raw coverage and *n* = base position.

We found a window of 25 bp gave the best compromise between improved signal to noise and mapping resolution after deconvolution for our data sets. This said, we later found that for the datasets analyzed here, smoothing did not give significant improvements in the final profile, but may still help in cases of low read density. See *Materials and Methods* for more detail.

Given that the rDNA unit is present ∼200 times in the biological mouse and human haploid genomes (Jackson *et al.* 2000; Henderson *et al.* 1972, 1974), and several rDNA pseudogene fragments are present in the annotated mouse *in silico* genome, we investigated the effects of permitting Bowtie 2 to report multiple alignments for each sequence read. The −*k* Reporting Mode parameter in Bowtie2 defines the number of genomic matches that are reported in the final alignment. We compared the alignments generated allowing only unique matches with those when up to three or nine matches were allowed (−*k 1*, *3*, and *9*) (Figure 2A). Improvements in mapping between −*k 1 and 3* were small (Figure 2, B and C, tracks 4a, b, and c), but, in some regions of the rDNA, such as over the enhancer repeats, UBF mapping became more uniform, consistent with the expected binding of this factor (Putnam and Pikaard 1992; Hamdane *et al.* 2014). Increasing −*k* to *9*, gave little further improvement. Since increasing the −*k* parameter in Bowtie2 also proportionately increased the computing time and the size of the resultant files, we set −*k* to *3* for all alignments.

The overall improvement in factor mapping using the deconvolution protocol can be qualitatively judged by comparing UBF binding across the enhancer repeats as computed using Bowtie2 or the same alignment followed by the deconvolution protocol (Figure 2, B–D). For example, a peak of UBF binding positioned over the Spacer, and 47S promoters was only convincingly observed after deconvolution (Figure 2D).

### Reproducibility of deconvoluted factor-binding profiles

**Figure 3:**
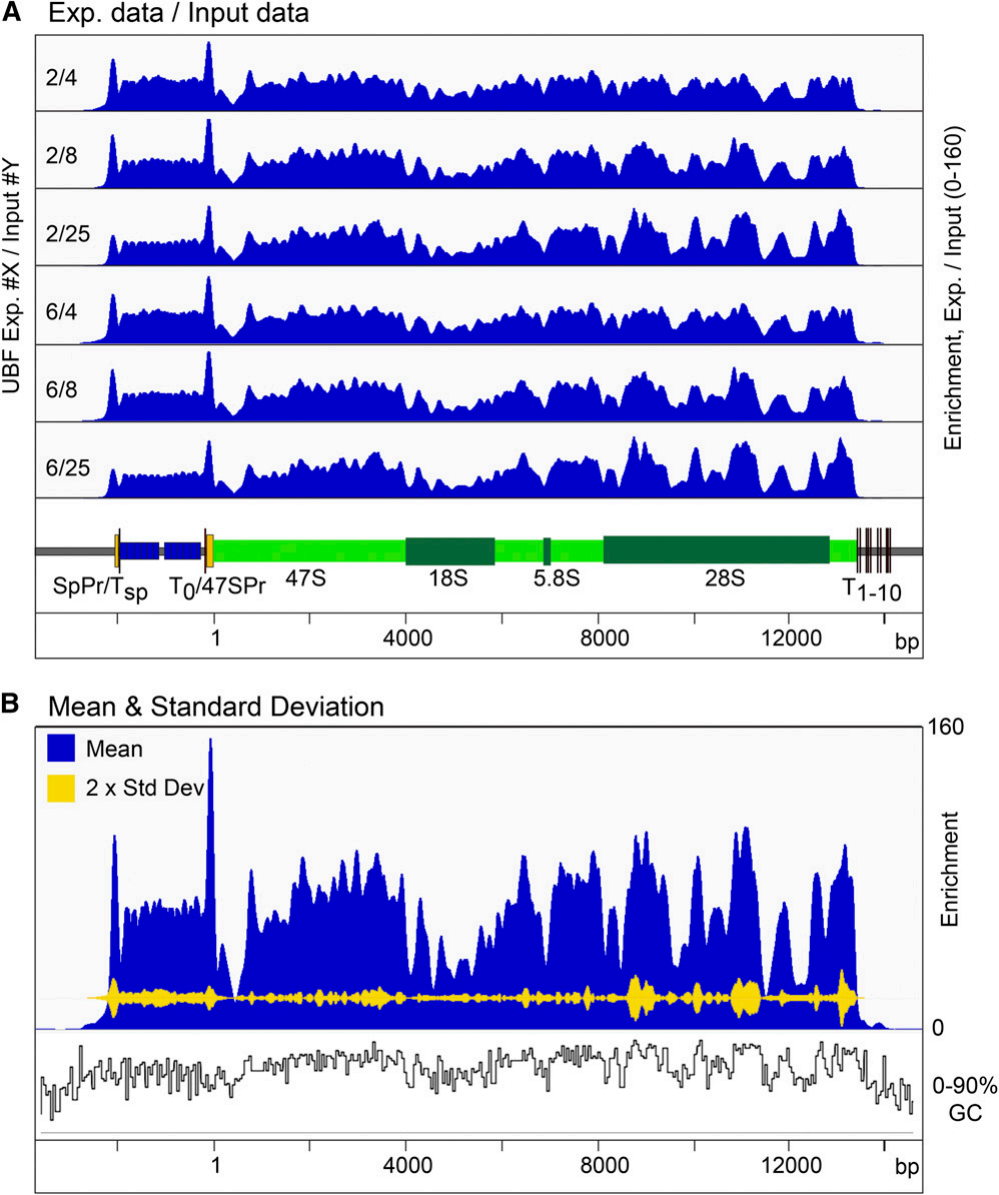
ChIP-Seq deconvolution maps are highly reproducible. (A) Comparison of two biological replicates of UBF ChIP-Seq data deconvoluted using data from three biological replicate “input” DNAs. (B) Mean coverage from the six deconvolutions in (A) is shown in blue and their SD in yellow. The vertical scale in (A and B) gives the enrichment relative to input DNA. A diagrammatic map of the rDNA is given below the mapping profiles in (A). The percent G+C sequence composition across the rDNA is also shown in (B).

To determine the degree of reproducibility of factor binding deduced using the deconvolution protocol, we compared the binding profiles obtained from different combinations of ChIP-Seq and input DNA biological replicates. Figure 3A shows each of two UBF ChIP-Seq replicates deconvoluted using sequence coverage obtained from three independent input DNA samples. Small variation in binding profile can be detected, but the overall distribution of UBF is essentially the same in all six calculations. This can be best judged when the SD between these data sets is plotted against the mean binding profile from all six (Figure 3B). Here it can be seen that the variability between the profiles is ≤10%, and small enough that, for most purposes, it can be neglected.

### UBF positioning over the 47S transcribed region is not random

UBF bound almost continuously throughout the 47S transcribed region, but, even after deconvolution, the interaction profile was much less uniform than that of RPI (cf. Figure 2, B and C), suggesting a nonrandom positioning of this factor. Over the 47S transcribed region the mean UBF profile followed the local GC content of the rDNA (Figure 3B), and the coefficient of determination *R*^2^ between these profiles of 0.47 indicated significant correlation (Supplemental Material, Figure S1A). This strongly suggested that the peaks and troughs of the UBF interaction profile resulted, at least in part, from a preferential positioning of this factor. We counted ∼74 peaks of UBF enrichment within the 47S transcribed region (Figure S1B), and these peaks displayed a mean spacing of 170 ± 58 bp. This was roughly consistent with the measured DNA contact length of a UBF dimer (Stefanovsky *et al.* 1996; Bazett-Jones *et al.* 1994), see *Discussion*.

### Applying deconvolution ChIP-Seq to map the mouse rDNA Spacer Promoter

**Figure 4:**
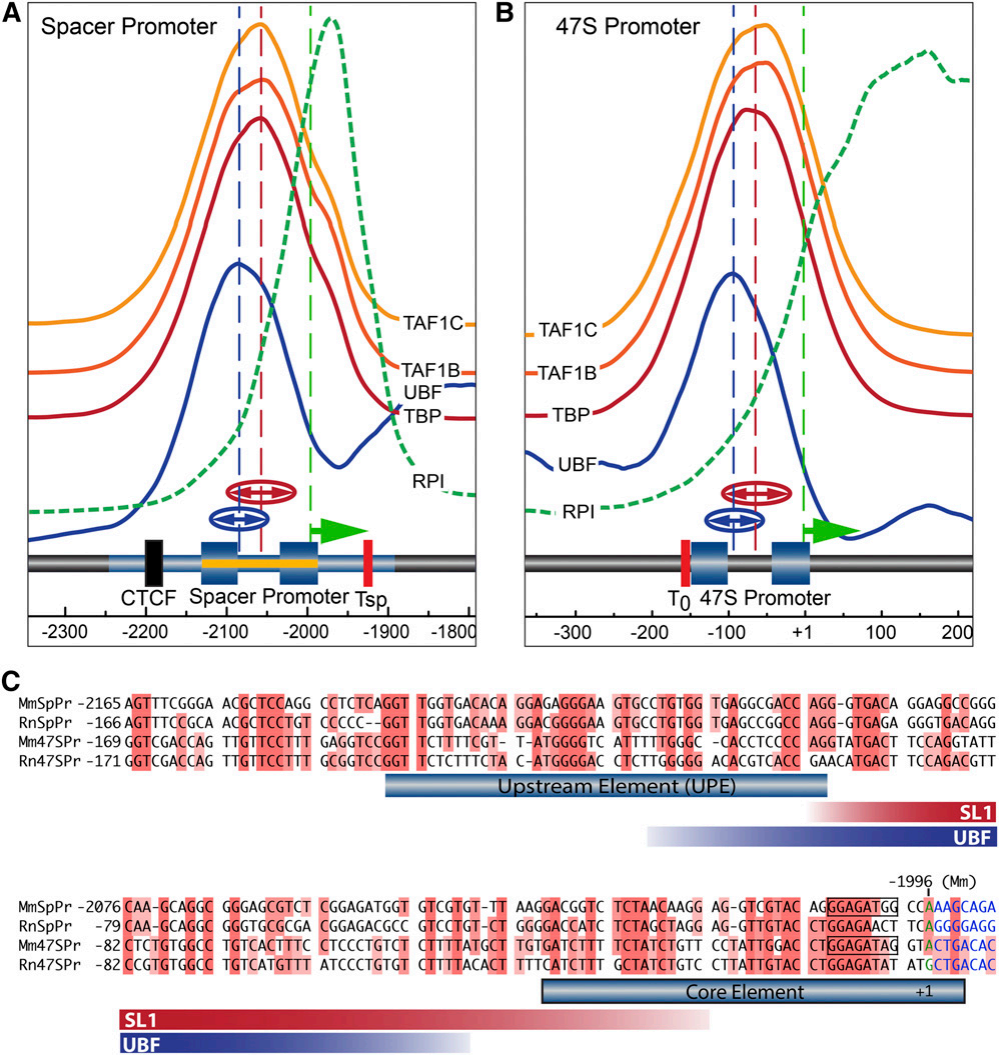
Mapping of preinitiation complexes at the Spacer and 47S Promoters of MEFs. (A and B) Show the interaction profiles of the TAF1B, -C, and TBP components of SL1, and of UBF and RPI across the Spacer and 47S Promoter regions in MEFs (MTAB-5893). The deconvoluted ChIP mapping profiles are shown stacked above a diagrammatic representation of the underlying rDNA sequence elements. The mapping profiles for each SL1 component (TAF1B, -C, and TBP) are shown on the same vertical scale of enrichment in (A and B), indicating that they are recruited equally efficiently at both promoters. For convenience, the vertical scale of enrichment for RPI at the two promoters is, however, different (see Figure 2B for a quantitative comparison). The extent of the Spacer Promoter was predicted by analogy to the 47S Promoter indicated by the blue-shaded boxes corresponding to the mapped UPE and Core elements. The original identification of the mouse Spacer Promoter and Spacer initiation site at −1996 bp (relative to the 47S initiation site, GenBank BK000964v3) (Kuhn and Grummt 1987), are indicated by blue shading band and an arrow (green). Functional mapping of the Spacer Promoter of rat (Smith *et al.* 1990), (−143 to +1 bp relative to the initiation site and requiring sequences upstream of −90 bp), is indicated in (A) by a yellow band. The broken vertical blue and red lines in (A and B) indicate the mean centers and “(↔)” the half-height half-widths of best-fit Gaussian distributions to the UBF and SL1-component mapping profiles obtained, respectively, from five to eight independent biological replicas. (C) Alignment of mouse (Mm) and rat (Rn) 47S and Spacer (SpPr) Promoters. The extent of SL1 components and UBF interactions are indicated by red and blue bands showing the mean half-height half-widths of the best-fit Gaussians to the mapping data, as in (A and B).

A functional Spacer Promoter was shown to lie within a 350 bp region of the mouse IGS (−2279 to −1930 bp relative to the 47S initiation site in GenBank BK000964v3) (Kuhn and Grummt 1987). In a cell-free assay, the transcription initiation site was mapped to −1996 bp adjacent to an imperfect 16 bp homology with the 47S Promoter (Figure 4C). However, nothing further is known of the structure of this Spacer Promoter, nor is it known whether it has the bipartite structure common to all major RPI promoters. The improved resolution of deconvolution ChIP-Seq allowed us to ask if binding of the preinitiation complex factors at the 47S and Spacer Promoters were similar, and to use this information to better map the Spacer Promoter. We identified binding peaks for three components of the SL1 complex (TAF1B, -C, and TBP) and for UBF at both promoters (Figure 4, A and B). The SL1 components displayed highly reproducible and exactly overlapping peaks of binding, strongly suggesting that, *in vivo*, they indeed bound as a complex as was expected (Moss *et al.* 2007). Gaussian peak-fit analysis showed that SL1 binding at the 47S and Spacer Promoters was centered, respectively, at 60 ± 1.2 and 65 ± 2.7 bp upstream of the corresponding initiation sites (vertical dashed lines in Figure 4, A and B). The position of the main peak of UBF interaction at each promoter was also highly reproducible, and was centered respectively at 83 ± 2.3 and 91 ± 2.2 upstream of the 47S and Spacer initiation sites. Thus, the peak of UBF binding was shifted upstream of the peak of SL1 binding by close to 20 bp at both promoters. The near identical positions of SL1 and UBF relative to the transcription initiation sites, see Figure S2 for an overlay, strongly argued that very similar, if not identical, preinitiation complexes formed at both 47S and Spacer Promoters. Further, the enrichment of each SL1 component and of UBF was found to be essentially identical at 47S and Spacer promoters, (note; the vertical enrichment scales are the same in Figure 4, A and B). It was concluded that, despite the extremely poor DNA base sequence homology between the two promoters (Figure 4C), UBF and SL1 must nonetheless recognize a common underlying promoter structure. Indeed, Marilley and Pasero (1996) predicted that rDNA promoters contain common features of curvature, twist, and helix stability that could explain their specific recognition by the transcription machinery.

### Deconvolution ChIP-Seq also identifies a Spacer Promoter within the human rDNA

**Figure 5:**
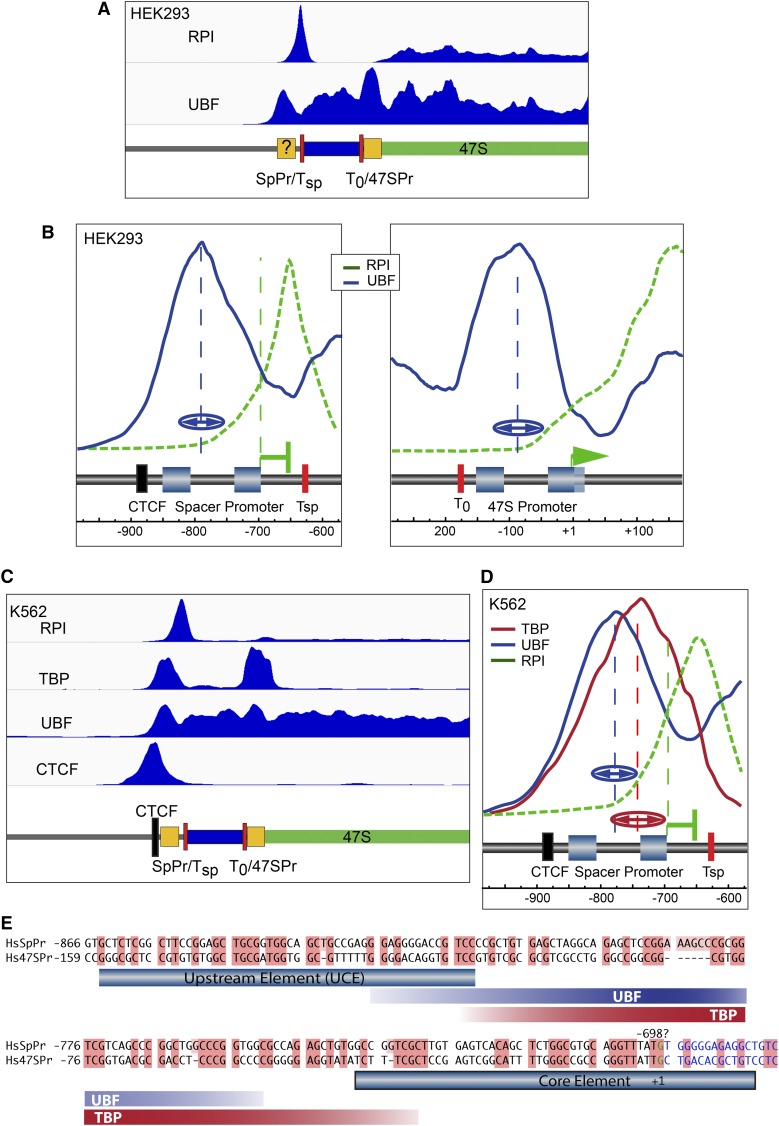
Identification of a Spacer Promoter in the human rDNA. (A) Deconvolution map of ChIP-Seq data for RPI and UBF (SRR087747, SRR087746, and SRR087753) (Zentner *et al.* 2011) across the 47S rRNA start site of the HEK 293T cell line. (B) High resolution plots of data in (A) over the 47S and prospective Spacer Promoter regions. A very similar arrangement to that in mouse is observed, with a peak of RPI lying ∼40 bp downstream of the predicted Spacer Promoter initiation site, and ∼20 bp upstream of the adjacent TTF1 binding site motif “T_sp_.” The identified 47S Promoter sequence motifs (Haltiner *et al.* 1986), the probable extent of the Spacer Promoter, and positions of the CTCF and TTF1 binding sites are indicated diagrammatically. (C) Realignment and deconvolution of ChIP-Seq data for RPI, TBP UBF, and CTCF (data sets; SRR502378/9, SRR2096736/7, E-MTAB-6032) from the human K562 cell line. The mapped and predicted sequence motifs are shown diagrammatically below the sequence coverage maps. (D) Detailed profiles of TBP, UBF, and RPI mapping at the human Spacer Promoter in K562 cells, (data sets; SRR770743-5, E-MTAB-6032), show a very similar arrangement to those in mouse and in HEK293T cells. Here again, a peak of RPI is detected downstream of the predicted initiation site and upstream of the adjacent TTF1 binding site motif “T_sp_.” The broken vertical blue and red lines in (B and D) indicate the mean centers, and “(↔)” the half-height half-widths of best-fit Gaussian distributions to the UBF and TBP mapping profiles obtained, respectively, from three to two independent biological replicas. (E) Alignment of human (Hs) 47S and predicted Spacer (SpPr) Promoter sequences. The extent of TBP and UBF interactions are indicated by red and blue bands showing the mean half-height half-widths of the best-fit Gaussians to the mapping data, as in (B and D).

Given its potential importance, it is surprising that a Spacer Promoter has not yet been identified in the human rDNA repeat, though references to its possible existence have been made in the literature (*e.g.*, Zentner *et al.* 2011; van de Nobelen *et al.* 2010). When we applied deconvolution ChIP-Seq to public datasets for UBF and RPI in human HEK cells, a peak of UBF binding was resolved near the mapped 47S Promoter, and at a site within the IGS ∼800 bp upstream of the 47S initiation site (Figure 5, A and B). UBF binding at the human 47S promoter was centered ∼90 bp upstream of the 47S initiation site, and so mapped much as in mouse (Figure 4B). Assuming the human 47S and Spacer Promoters have similar organization, we were able to make an initial estimate of the position of the human Spacer Promoter as between −850 and −700 bp relative to the 47S initiation site.

Deconvolution analysis of public and in-house ChIP-Seq data for RPI, TBP, and UBF from human K562 cells further supported this Spacer Promoter mapping. Two peaks of TBP binding were observed on the rDNA, one at the 47S promoter and the other over the prospective Spacer Promoter site, and each TBP peak corresponded to a peak in the UBF binding profile (Figure 5C). At higher resolution, it was seen that each TBP peak in fact mapped ∼30 bp downstream of the corresponding peak of UBF (*e.g.*, Figure 5D), suggesting a very similar promoter organization to that in mouse. Gaussian curve fitting to the binding profiles from both HEK and K562 cells placed the mean peak centers for TBP and UBF at the prospective Spacer Promoter at −758 ± 12 and 789 ± 8, respectively, relative to the 47S initiation site, while, at the 47S Promoter, mean peak centers for TBP and UBF were −78 ± 16 and −87 ± 3. Assuming a similar positioning of TBP and UBF relative to the initiation sites at both promoters, this places the Spacer Promoter initiation site at −691 ± 11. Alignment of the two promoter sequences shows a potential homology in this region, suggesting that the Spacer Promoter initiates transcription at or near −698 bp (Figure 5E).

### The chromatin contexts of the human and mouse Spacer Promoters are very similar

We previously found that, in mouse, RPI transcription initiated at the Spacer Promoter is arrested ∼40 bp downstream, adjacent to the binding site for the RPI Transcription Termination Factor TTF1 (Hamdane *et al.* 2014; Herdman *et al.* 2017) (Figure 4A). Strikingly, a peak of RPI was also observed just 50 bp downstream of the probably human Spacer Promoter, and immediately adjacent to a consensus binding site (GGTCGACC) for TTF1 (Figure 5B). This striking similarity between the two systems strongly suggested that, not only did the human rDNA possess an active Spacer Promoter, but that it was also regulated by TTF1 in a very similar manner. A further characteristic of the mouse Spacer Promoter was its position adjacent to a unique boundary complex consisting of CTCF, and an upstream concentration of active chromatin marks (Herdman *et al.* 2017). Screening the sequenced human 43 kbp rDNA repeat unit for likely CTCF binding sites using CTCFDSDBv2.0 (Ziebarth *et al.* 2013) revealed four potential sites with log-odd scores (Altschul *et al.* 2010) ∼14, and one immediately upstream of the prospective Spacer Promoter (−896 to −876) with a log-odd score of over 19, (that is 80 × more likely than random). As previously shown (Zentner *et al.* 2011), alignment of public CTCF ChIP-Seq data from K562 cells revealed a single site of interaction corresponding to this best predicted CTCF site (Figure 5C). Thus, the chromatin and RPI factor contexts strongly suggest that not only have we accurately identified an active Spacer Promoter in the human rDNA, but also that it forms part of an entity analogous to the enhancer boundary complex recently identified in mouse rDNA (Herdman *et al.* 2017).

### A common mode of TBP-complex binding at the human spacer and 47S promoters

**Figure 6:**
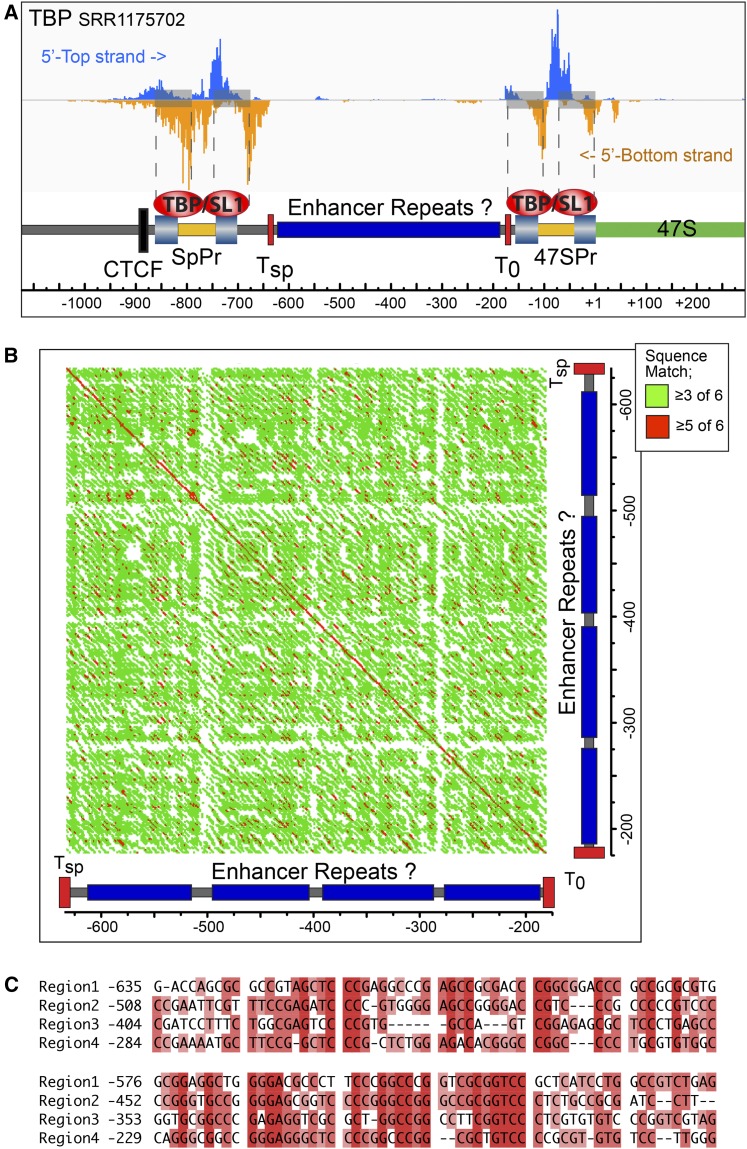
Fine mapping of TBP complexes and potential enhancer repeat suggest functional parallels between the human and mouse rDNA. (A) Realignment of TBP ChIP-exonuclease data from human K562 cells (He *et al.* 2015; GEO Acc. GSE55306), onto the human rDNA reveals dual contact sites at both Spacer and 47S Promoters. Spacer and 47S Promoter Core and UPE are indicated by light blue shaded boxes and potential T_sp_ and T_0_ TTF1 sites by red boxes. (B) “DotPlot” homology alignment of human rDNA sequences lying between the Spacer and 47S Promoters generated using the Gene Inspector software (Textco) and a sliding window of six bases. Red indicates ≥5/6 identically matching bases, and green ≥3/6 matches. (C) Alignment of the four pseudorepeats within the same region. In (A–C) sequence position is indicated relative to the 47S initiation site.

We took advantage of available ChIP-exonuclease mapping data for TBP in K562 (He *et al.* 2015) to better define SL1 complex interactions on the human rDNA. Realignment of the raw data revealed the potential 5′ and 3′ boundaries of the TBP-containing complexes (5′-top and 5′-bottom in Figure 6A). The data clearly identified complexes at both 47S and Spacer Promoters, and suggested two DNA contact sites within each promoter. Strikingly, the sites corresponded closely to the mapped UPE (UCE) and core promoter elements of the human 47S promoter (Haltiner *et al.* 1986), and suggested that the SL1 complex either contacts both promoter elements or that mammalian rDNA promoters, like the yeast rDNA promoter, recruit two distinct TBP associated complexes (Moss *et al.* 2007), see *Discussion*. The ChIP-exonuclease data further reinforce the notion that, despite the poor primary sequence conservation, the 47S and Spacer Promoters have very similar binary structures.

### Identification of potential enhancer repeats in the human rDNA

The DNA lying immediately upstream of the major rRNA promoter in a wide range of eukaryotes has been found to include a variable number of short (∼60–200 bp) sequence repeats (Moss *et al.* 1985, 2007). In *Xenopus* and mouse these repeats possess enhancer or selector-like activities (Moss 1983; Labhart and Reeder 1984; De Winter and Moss 1986, 1987; Pape *et al.* 1989; Pikaard *et al.* 1990; Osheim *et al.* 1996; Moss *et al.* 2007). Our mapping of the human Spacer Promoter allowed us to investigate the organization of sequences within the region lying between it and the 47S Promoter. Though we found no clear evidence for near perfect “enhancer-like” repeats, a “DotPlot” search for homologies did reveal evidence for an underlying repetition of short highly GC-rich sequence homologies interspersed at roughly 100 bp intervals by more complex sequence (Figure 6B). Alignment of these “repeat” units suggested that they possibly have a common evolutionary origin, and so may indeed be analogous to the enhancer repeats seen in other organisms (Figure 6C). Analysis of more recent rDNA sequences (GB Acc. AL3536449, AL592188, FP236383, and KC876030) also suggest that, unlike the rDNA of many other organisms, this region of the human rDNA is fully conserved, showing at most a 10 bp length difference with the most commonly referenced composite rDNA repeat sequence (GB Acc. U13369.1). This said, it should be noted that these newer sequences originate from Bacmids containing the rDNA Nucleolar Organizer Region (NOR) boundaries from specific chromosomes, and so may not be representative of the bulk rDNA.

## Discussion

The potential for very significant improvements in ChIP-Seq mapping resolution afforded by our simple deconvolution protocol were recently demonstrated when the protocol was applied to map transcription factors and chromatin status across the mouse rDNA (Herdman *et al.* 2017). Here, we provide a detailed deconvolution protocol, consider the effects of data smoothing and multiple site alignment, and demonstrate the reproducibility of the interaction maps generated. We show that, given sufficient sequencing depth, variations in mapping profiles are small (±10%), and may, in large, part represent the variability introduced by the ChIP protocol and/or by biological variability between samples. In principle, our deconvolution protocol is applicable to any ChIP-Seq data for which sufficient sequencing depth is available. Based on our present studies, we estimate that the average number of reads across each base position of both input and ChIP datasets needs to be ≥100 in order for the deconvolved profiles to be statistically significant. Such a situation is easily attainable with present sequencing technologies.

When applied to ChIP-Seq data for the RPI polymerase, the deconvolution protocol revealed a near uniform recruitment across the 47S transcribed region of the mouse rDNA. In contrast, the recruitment of UBF across the same region displayed ∼74 preferential positions spaced on average at 170 bp intervals. Closer inspection also revealed a correlation between UBF binding and the GC content of the underlying rDNA. Previous analyses have shown that UBF has a preference for GC-rich DNA (Copenhaver *et al.* 1994), and that a UBF dimer interacts with 110–160 bp of DNA, looping it into a single turn and leading to the suggestion that it may replace nucleosomal chromatin (Stefanovsky *et al.* 1996; Bazett-Jones *et al.* 1994; Herdman *et al.* 2017). Together, the data suggest that UBF dimers bind at preferential sites to form a semicontinuous pseudochromatin across the 47S transcribed region of the rDNA.

We have also applied the deconvolution protocol to fine map the 47S and Spacer Promoters of the mouse and human rDNA IGSs. Interestingly, the data suggest that, despite a complete lack of any significant homology at the level of the respective DNA sequences, the structure and the chromatin contexts of the human and mouse Spacer Promoters are very similar. We found that positioning of the preinitiation factors, UBF and the components of the RPI TBP complex SL1, is nearly identical at the 47S and Spacer Promoters in both mouse and human. Further, we found that the ChIP enrichment of the known SL1 subunits at 47S and Spacer Promoters was, within experimental error, the same. Thus, all active rDNA units appear to recruit SL1 at both promoters with equal efficiency.

In contrast, the context of the Spacer Promoters, in being flanked immediately upstream by CTCF and Cohesin complexes and downstream by an arrested polymerase, is quite different from that of the 47S Promoter. As we recently demonstrated in mouse, the CTCF complex forms a boundary between the upstream chromatin and the transcriptionally active rDNA unit (Herdman *et al.* 2017). Loss of CTCF was also shown to eliminate UBF recruitment to the rDNA (van de Nobelen *et al.* 2010). Thus, the CTCF boundary most probably arrests the expansion of upstream repressive chromatin into the active rDNA unit. The recruitment of the Snf2h chromatin remodeller subunit at the CTCF site is probably important in this respect (Herdman *et al.* 2017). Recruitment of Cohesin to the CTCF boundary further suggests a role in chromatin looping and the spatial organization of the rDNA loci, see Herdman *et al.* (2017) for further discussion.

The Spacer Promoter is also unique in being associated with a strong interaction peak of RPI. This peak is centered downstream of the initiation site and upstream of the adjacent TTF1 binding site, and suggests that transcription from this promoter is arrested after only 40–50 nucleotides in both mouse and human. Release of this arrested polymerase into active elongation would generate a long noncoding RNA (lncRNA) that has been suggested to control *in trans* rDNA silencing in mouse (Savic *et al.* 2014). It could potentially also regulate the activity of the mouse enhancer repeats lying downstream. Analysis of the sequences lying between the Spacer Promoter and 47S Promoter suggested that enhancer repeats may also exist in this region of the human rDNA, and, hence, could quite possibly be analogous in function to the mouse and *Xenopus* enhancers. But, a demonstration of this must await functional studies.

While the RPI promoters of different organisms from yeast to human show little or no DNA sequence conservation, they do conserve a common functional organization of precisely spaced UPE and core elements, suggesting a similar mode of recognition by the transcription machinery. In fact, we found that realignment of the ChIP-exonuclease (ChIP-nexus) data for TBP (He *et al.* 2015) revealed two distinct contact sites for SL1 that mapped closely to the UPE (UCE) and core promoter elements of the human 47S promoter (Haltiner *et al.* 1986), see Figure 6A. This suggested either that a single SL1 complex contacts both promoter elements or that, as we have previously suggested, mammalian rDNA promoters, might recruit two SL1 complexes (Moss and Stefanovsky 2002). However, whether these contact sites would correspond to two identical SL1 complexes, or to two SL1 subcomplexes as seen in yeast, where distinct TAF1 subcomplexes bind UPE and core elements and are bridged by TBP (Moss *et al.* 2007), will require further study. It is relevant here to note that our present knowledge of the structure of mammalian SL1 is still incomplete (Gorski *et al.* 2007; Murano *et al.* 2014).

## Supplementary Material

Supplemental material is available online at www.g3journal.org/lookup/suppl/doi:10.1534/g3.117.300225/-/DC1.
