# Supplementary material for: A Deconvolution Protocol for ChIP-Seq Reveals Analogous Enhancer Structures on the Mouse and Human Ribosomal RNA Genes
Source: G3 (Bethesda). 2017 Nov 20;8(1):303–14. doi: 10.1534/g3.117.300225 (PMC5765358; doi:10.1534/g3.117.300225)
Supplement: Supplementary file 1 [file 303FigureS1.pdf]

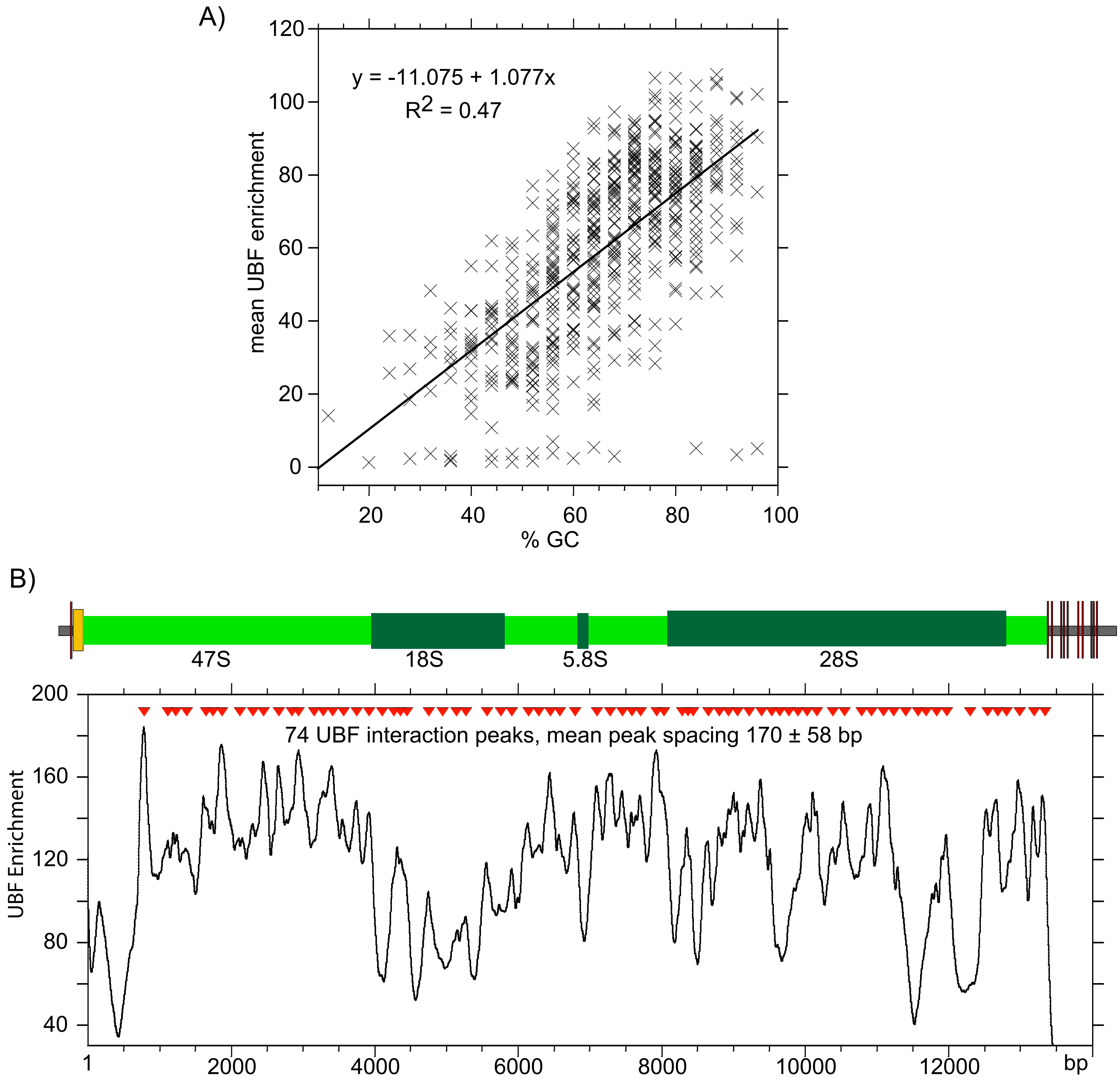

Figure S1. Preferential positioning of UBF across the mouse rDNA. A) The mean UBF enrichment data from Figure 3B is shown plotted against the rDNA GC content using 25 bp data windows. The least squares linear best fit and the corresponding coefficient of determination R2 are shown. B) Peak positions of UBF interaction across the 47S transcribed region were identified for a single deconvoluted UBF dataset (E-MTAB-5839, ChIP-seq_UBF_MEFs_UBFwt_4HT_Rep3.bedgraph). The mean spacing of peaks was calculated as 170 bp ± a standard deviation of 58 bp.
