# Supplementary material for: A Deconvolution Protocol for ChIP-Seq Reveals Analogous Enhancer Structures on the Mouse and Human Ribosomal RNA Genes
Source: G3 (Bethesda). 2017 Nov 20;8(1):303–14. doi: 10.1534/g3.117.300225 (PMC5765358; doi:10.1534/g3.117.300225)
Supplement: Supplementary file 2 [file 303FigureS2.pdf]

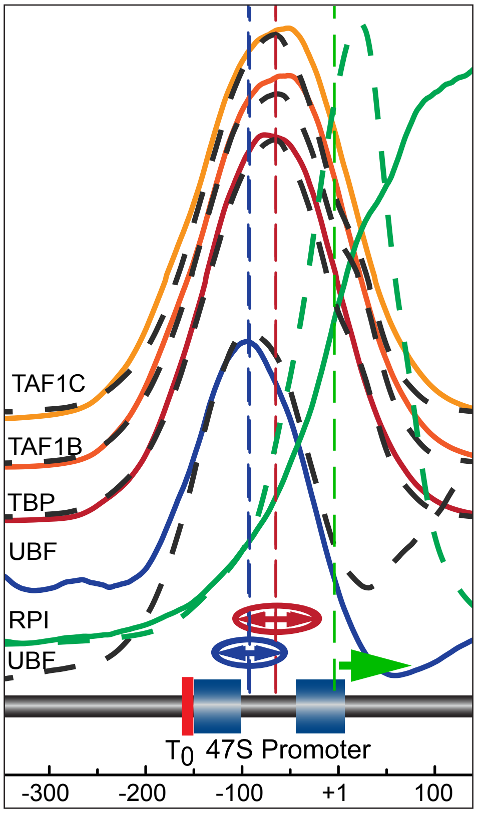

Figure S2. Direct comparison of interaction profiles of the TAF1B, -C and TBP components of SL1, and of UBF and RPI at the Spacer and 47S Promoter regions in MEFs (MTAB-5893). The data from Figure 4A and B are shown superimposed, and the 47S and Spacer Promoter initiation sites are aligned. The profile colouring is as in Figure 4, except that the Spacer Promoter profiles corresponding to each SL1 component and to UBF are shown as dashed black lines. The RPI profiles have been included in green, that at the Spacer Promoter as a dashed line. The broken vertical blue and red lines indicate the mean centres and "**<->**" the half-height half-widths of best-fit Gaussian distributions to the UBF and SL1-component mapping profiles at both promoters and superimpose exactly.
